# Novel Mad2-targeting miR-493-3p controls mitotic fidelity and cancer cells’ sensitivity to paclitaxel

**DOI:** 10.18632/oncotarget.7860

**Published:** 2016-03-02

**Authors:** Mahesh Tambe, Sofia Pruikkonen, Jenni Mäki-Jouppila, Ping Chen, Bente Vilming Elgaaen, Anne Hege Straume, Kaisa Huhtinen, Olli Cárpen, Per Eystein Lønning, Ben Davidson, Sampsa Hautaniemi, Marko J. Kallio

**Affiliations:** ^1^ Department of Physiology, Institute of Biomedicine, University of Turku, Turku, Finland; ^2^ Centre for Biotechnology, University of Turku, Turku, Finland; ^3^ Department of Pharmacology, Drug Development and Therapeutics, University of Turku, Turku, Finland; ^4^ Drug Research Doctoral Programme and FinPharma Doctoral Program Drug Discovery, Finland; ^5^ Turku Doctoral Program of Molecular Medicine, University of Turku, Turku, Finland; ^6^ Research Programs Unit, Genome-Scale Biology, Faculty of Medicine, University of Helsinki, Helsinki, Finland; ^7^ Department of Gynecological Oncology, Oslo University Hospital, Norwegian Radium Hospital, Oslo, Norway; ^8^ Department of Clinical Science, University of Bergen and Department of Clinical Oncology, Haukeland University Hospital, Bergen, Norway; ^9^ Department of Pathology, University of Turku and Turku University Hospital, Turku, Finland; ^10^ Auria Biobank, Turku, Finland; ^11^ Department of Pathology, Oslo University Hospital, Norwegian Radium Hospital, Oslo, Norway; ^12^ Institute of Clinical Medicine, Faculty of Medicine, University of Oslo, Oslo, Norway

**Keywords:** miR-493-3p, Mad2, spindle assembly checkpoint, aneuploidy, taxane resistance, Chromosome Section

## Abstract

The molecular pathways that contribute to the proliferation and drug response of cancer cells are highly complex and currently insufficiently characterized. We have identified a previously unknown microRNA-based mechanism that provides cancer cells means to stimulate tumorigenesis *via* increased genomic instability and, at the same time, evade the action of clinically utilized microtubule drugs. We demonstrate miR-493-3p to be a novel negative regulator of mitotic arrest deficient-2 (*MAD2*), an essential component of the spindle assembly checkpoint that monitors the fidelity of chromosome segregation. The microRNA targets the 3′ UTR of Mad2 mRNA thereby preventing translation of the Mad2 protein. In cancer cells, overexpression of miR-493-3p induced a premature mitotic exit that led to increased frequency of aneuploidy and cellular senescence in the progeny cells. Importantly, excess of the miR-493-3p conferred resistance of cancer cells to microtubule drugs. In human neoplasms, miR-493-3p and Mad2 expression alterations correlated with advanced ovarian cancer forms and high miR-493-3p levels were associated with reduced survival of ovarian and breast cancer patients with aggressive tumors, especially in the paclitaxel therapy arm. Our results suggest that intratumoral profiling of miR-493-3p and Mad2 levels can have diagnostic value in predicting the efficacy of taxane chemotherapy.

## INTRODUCTION

Despite the great advancement of personalized medicine to treat cancer, the microtubule dynamics targeting drugs such as taxanes, epothilones and vinca alkaloids still remain the major weapons against ovarian, cervical, breast and non-small cell lung cancer. Unfortunately, clinical responses to these front line chemotherapeutics are highly variable and acquired resistance is a common event. These caveats stem partly from the poor understanding of the molecular pathways that confer microtubule drug sensitivity or resistance in cancer cells. As a result, it is currently nearly impossible to predict in advance which patients will benefit from the microtubule drug therapy. MicroRNAs (miRNAs) are short non-coding RNA molecules that have been implicated as biomarkers of drug efficacy and drug safety as well as to hold promise for improved disease diagnostics.

MiRNAs function by binding to the complementary sequences in target gene mRNAs, thereby suppressing gene expression through translational inhibition and/or mRNA destabilization. A majority of miRNA targeting sites reside in the 3′ untranslated region (3′ UTR) of the target mRNA [1]. A miRNA is transcribed either from an intron of protein-coding gene or from an independent transcription unit [1, 2]. miRNAs are estimated to regulate more than half of human protein-coding genes, and thus have the potential to influence a wide spectrum of cellular pathways ranging from cell survival and apoptosis to development and differentiation. Due to their role as regulators of central physiological processes, altered miRNA expression contributes to a variety of pathological states, including cancer [3-5]. Regardless of the growing interest towards miRNAs and their role in health and disease, very little is known about miRNA-mediated control of mitosis and microtubule drug sensitivity.

During mitosis, a regulatory signal transduction pathway called the Spindle Assembly Checkpoint (SAC) ensures equal distribution of genetic material between the forming daughter cells [6, 7]. The SAC is triggered during early mitosis and stays active until all chromosomes in the cell have established stable bipolar connections to spindle microtubules and are aligned to the cell equator. Mad2 (*MAD2L1*) is one of the key SAC proteins responsible for transduction of the anaphase inhibitory signal emanating from unattached kinetochores of chromosomes [8].

In early mitosis, Mad2 builds up the Mitotic Checkpoint Complex (MCC) together with Bub3, BubR1 and Cdc20. MCC restrains the ability of Cdc20 to activate the Anaphase Promoting Complex/Cyclosome (APC/C), a mitotic E3 ubiquitin ligase that is responsible for directing anaphase inhibitor proteins for proteasome-mediated destruction [7, 9, 10], thereby preventing sister chromatid separation and anaphase onset [11]. Upon proper chromosome alignment, the MCC disassembles allowing activation of APC/C and cell cycle progression into anaphase [12]. For these reasons, Mad2 is indispensable for the activation of the SAC and maintenance of genomic balance [7, 8].

Altered expression of SAC proteins and the consequent defects in the checkpoint function have been linked to promotion of aneuploidy *via* erroneous chromosome segregation [13]. In case of Mad2, cells and animals with deregulated Mad2 levels exhibit serious mitotic anomalies giving rise to chromosomal instability (CIN) and promotion of tumour progression [14, 15] and tumour relapse [16]. Moreover, low intratumoral Mad2 levels correlate with poor prognosis and lower recurrence-free survival rates in cancer patients [17, 18]. Interestingly, mutations of the Mad2 gene are rare in cancer [19, 20] which further emphasizes the importance of proper control of Mad2 expression for normal growth. The mechanisms capable of causing changed Mad2 expression during tumorigenesis are many and include, for example, haploinsufficiency by genomic rearrangements and altered gene dosage, epigenetic gene inactivation, and defects in Mad2 production at transcriptional or translational level. For example at the transcriptional level, overexpression of Mad2 protein has been observed upon adenovirus E1A -mediated inactivation of pRb, which causes stimulation of E2F-dependent transcription of Mad2 mRNA [21] while in cells with deregulated repressor-element-1-silencing transcription factor (REST) the Mad2 protein levels were found to be decreased [22]. In both cases, the aberrant Mad2 levels were associated with mitotic defects causing aneuploidy. Here we report the discovery of a novel post-transcriptional regulator of Mad2, miR-493-3p, and demonstrate how excess of the miRNA causes aneuploidy and development of microtubule drug resistance in cancer cells.

## RESULTS

### Excess miR-493-3p compromises microtubule drug induced M phase arrest and in drug-free culture accelerates mitosis

**Figure 1:**
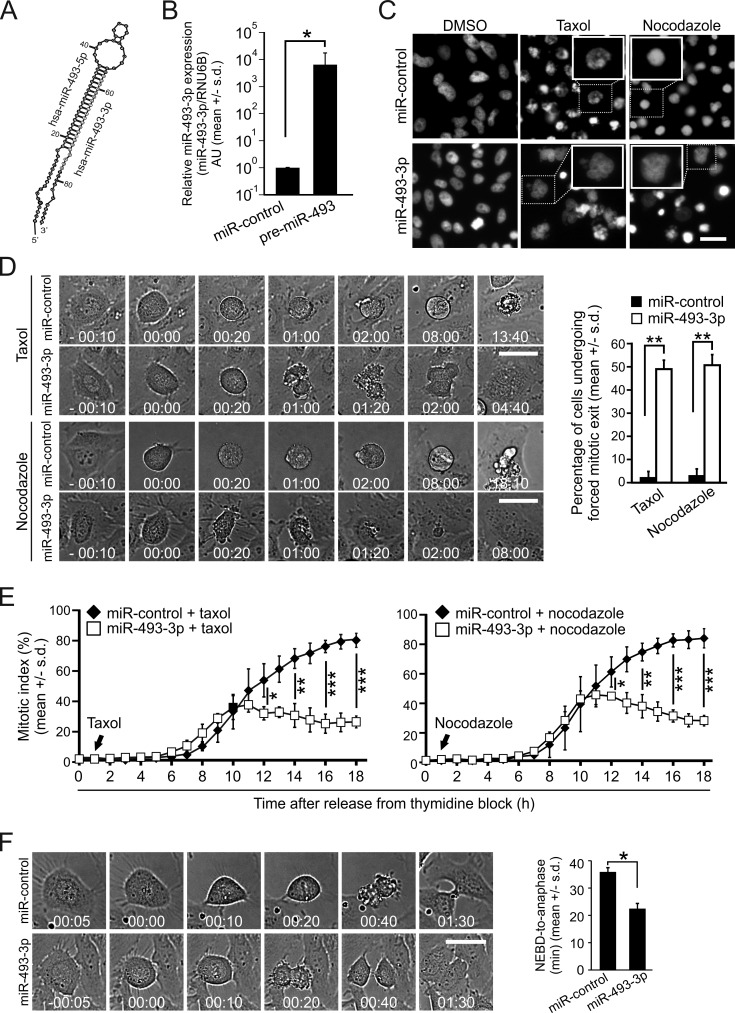
Excess of miR-493-3p weakens the SAC **A.** Schematic illustration of the miR-493 hairpin loop. **B.** Quantification of the miR-493-3p levels in HeLa cells 24 h after the transfection with miR-control or pre-miR-493. **C.** Representative fluorescence images of DAPI stained nuclei of miR-control and miR-493-3p overexpressing HeLa cells fixed after overnight treatment with taxol or nocodazole. Scale bar, 50μm. **D.** Representative still images of non-synchronized time-lapse filmed miR-control and miR-493-3p overexpressing HeLa cells treated with taxol or nocodazole (0 min = NEBD). Scale bar, 25 μm. The graph shows quantification of forced mitotic exit in miRNA transfected cell populations cultured in the presence of taxol or nocodazole (*n* = 300 cells per treatment). **E.** Quantification of mitotic indices of miRNA-transfected and synchronized HeLa cell populations after release from the thymidine block into taxol or nocodazole (n≥600 cells analyzed for each time point). **F.** Representative still images of non-synchronized miRNA-transfected HeLa cells undergoing mitosis in drug-free culture conditions (0 min = NEBD). Scale bar, 25 μm. The graph shows quantification of mitotic duration in the indicated miRNA transfected cells (*n* = 300 cells per group). All data is mean +/− s.d. from 3 independent experiments. The asterisks denote statistical significance (* = *p* ≤ 0.05, ** = *p* ≤ 0.01).

Mir-493-3p (Figure 1A-1B) was one of the hits from our cell-based high-throughput screen (HTS) for miRNAs that antagonize microtubule drug induced mitotic block [23]. The majority of HeLa cervical cancer cells overexpressing miR-493-3p evaded mitotic block induced by a microtubule stabilizing drug taxol or microtubule depolymerizing agent nocodazole, and formed large progeny cells with a multilobed nuclear morphology (Figure 1C). This was in contrast to cells transfected with non-targeting control miRNA (miR-control), which exhibited a long mitotic arrest with condensed chromosomes when treated with the drugs (Figure 1C). To confirm the result and visualize the timing of forced mitotic exit by miR-493-3p we monitored taxol or nocodazole treated miR-control or miR-493-3p transfected cell populations using time-lapse microscopy. As expected, majority of the microtubule drug treated miR-control overexpressing cells arrested at M phase for longer than 8 hours before they underwent cell death (Figure 1D). In contrast, many cells with excess miR-493-3p exhibited a forced mitotic exit within 100 minutes after entry to M phase despite the presence of taxol or nocodazole in the culture medium (Figure 1D). Quantification of the time-lapse films indicated that in response to taxol, an average of 49.0 +/− 4.4% of the mitotic cells in the miR-493-3p overexpressing cell populations underwent the forced mitotic exit, which is significantly more compared to the average of 2.0 +/− 2.6% in the miR-control transfected controls (*p* = 0.002, Figure 1D). Similar results were obtained with miRNA transfected and nocodazole treated cells (Figure 1D) as well as with synchronized HeLa cell populations that were released from G1/S block into growth medium containing taxol or nocodazole (Figure 1E). When cycling non-drug treated miR-control or miR-493-3p transfected HeLa cells were time-lapse filmed we noted a significant difference in the time the cells spent in mitosis (Figure 1E); the average time from nuclear envelope breakdown (NEBD) to onset of anaphase for the miR-control and miR-493-3p transfected cells was 35.7 +/− 1.7 min and 22.3 +/− 2.0 min, respectively (*p* = 0.02). Based on these results we conclude that excess miR-493-3p enables cells to escape spindle poison induced M phase block and in drug-free culture conditions accelerates mitosis.

### miR-493-3p targets the 3′UTR of Mad2 mRNA and suppresses Mad2 gene expression directly

**Figure 2:**
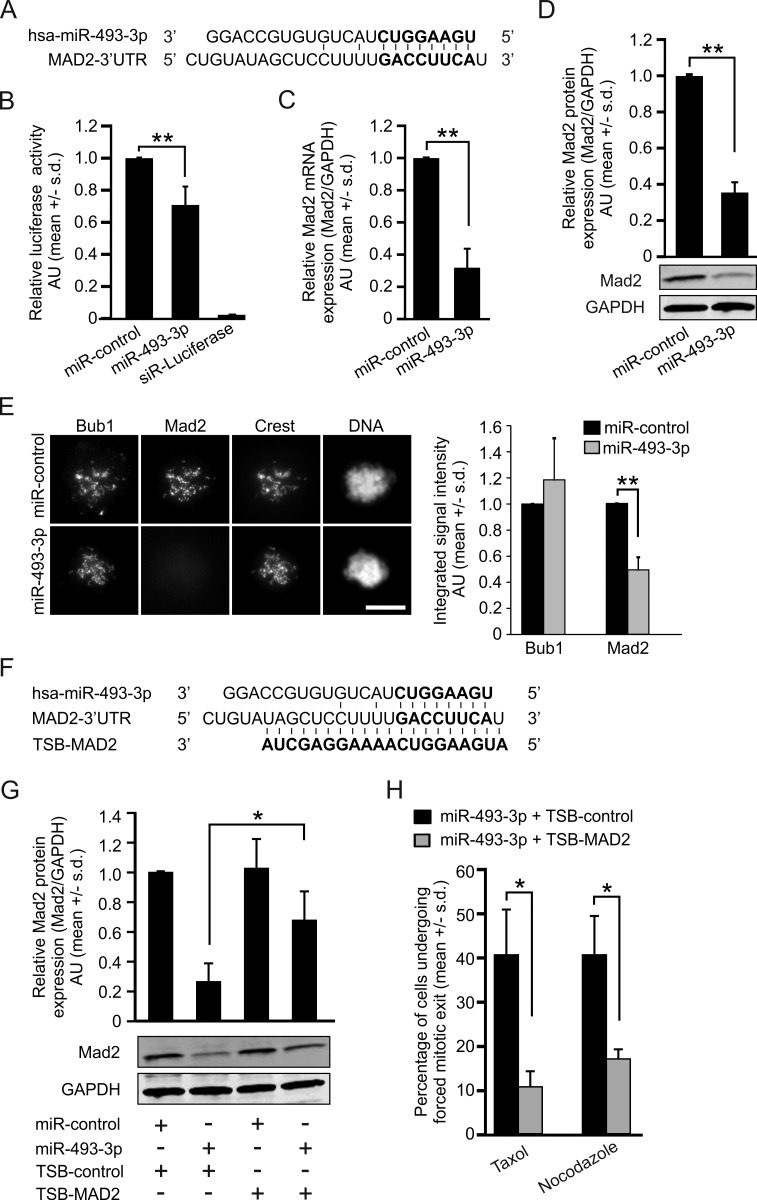
miR-493-3p targets the Mad2-3′UTR and downregulates Mad2 expression **A.** Schematic illustration of the predicted targeting site of miR-493-3p in the Mad2 mRNA 3′UTR. **B.** Quantification of relative firefly luciferase activity, normalized to Renilla luciferase activity, measured from HeLa cells 24 h after co-transfection of Mad2-3′UTR -firefly luciferase reporter construct and miRNA (miR-control or miR-493-3p). **C.** Quantification of Mad2 mRNA and **D.** protein levels at 48 h post-transfection in HeLa cells. A representative Western blot is shown. **E.** Representative immunofluorescence images showing the impact of miRNA overexpression on Mad2 and Bub1 labelling in prometaphase HeLa cells 48 h post-transfection. Crest served as a kinetochore marker and DNA was stained with DAPI. Scale bar, 10 μm. The graph shows quantification of Mad2 and Bub1 at whole cell level (50 mitotic cells were analysed per a group). **F.** Sites of interference for miR-493-3p and Mad2-target site blocker (TSB-MAD2) in the Mad2-3′UTR (bold letters indicate the seed sequence). **G.** Quantification of Mad2 protein levels after indicated transfections. A representative Western blot is shown. **H.** Quantification of miRNA-transfected cells undergoing forced mitotic exit in the presence of taxol or nocodazole (*n* = 300 cells per group). All data shown is mean +/− s.d. from 3-4 independent experiments. The asterisks denote statistical significance (* = *p* ≤ 0.05, ** = *p* ≤ 0.01).

Earlier studies have shown that miR-493-3p participates in the regulation of cell motility and migration *via* targeting FZD4, RhoC, IGF1R and MKK7 [24-26]. To identify new miR-493-3p target genes implicated in control of mitosis, we performed a global gene expression analysis using miRNA transfected HeLa cells. One of the most striking gene expression alterations induced by excess miR-493-3p was downregulation of Mad2, which was reduced by 1.78 fold in the miR-493-3p overexpressing cells in comparison to miR-control (*p* = 0.0002, Table S1). Next, using the *in silico* prediction software TargetScan and micTar we found that the mRNA of Mad2 harbors a predicted miR-493-3p targeting sequence in its 3′UTR (Figure 2A). To determine whether miR-493-3p and Mad2 mRNA interact, we performed targeting assays using a firefly luciferase reporter gene construct containing the Mad2-3′UTR sequence. HeLa cells co-transfected with miR-493-3p and the Mad2-3′UTR luciferase reporter plasmid showed significantly reduced luciferase activity (*p* = 0.01) when compared to miR-control overexpressing cells (Figure 2B). Quantification of the readouts from qRT-PCR and Western blotting validated the suppression of Mad2 by excess miR-493-3p; both mRNA and protein levels of Mad2 were significantly reduced by an average of 68 +/−11% (*p* = 0.009) and 65 +/−5% (*p* = 0.002) in miR-493-3p overexpressing cells, respectively, in comparison to miR-controls (Figure 2C-2D). Similar results were obtained with human adenocarcinoma cells of colon (HCT-116) and breast (MCF7) origin (Figure S1). To confirm the suppression of Mad2 in individual mitotic cells by excess miR-493-3p we accumulated miR-control or miR-493-3p overexpressing HeLa cells to pre-anaphase by a 4 h nocodazole treatment and then immunostained the cells with an anti-Mad2 and anti-Bub1 antibodies. In the miR-493-3p transfected cells, Mad2 signal was significantly diminished in comparison to miR-control (down by 50.76 +/− 9.64%, *p* = 0.01, Figure 2E). Importantly, the amount of Bub1, which is required for the kinetochore location of Mad2 [27, 28] did not change significantly in the cells with excess miR-493-3p (elevated by 18.46 +/− 31.94%, *p* = 0.42, Figure 2E). Based on the results from *in-silico*, luciferase reporter and cell-based assays we conclude that miR-493-3p targets Mad2 for suppression.

### miR-493-3p impact on Mad2 expression is not due to E2F1 suppression or off-target effect

MiR-493-3p was recently reported to target E2F1 [29], which is a putative transcription factor of Mad2 [21]. To test this in our cell models, we investigated the impact of miR-493-3p overexpression on E2F1 mRNA and protein levels. In line with Gu et al. (2014), the E2F1 protein levels were significantly reduced in HeLa, HCT-116 and MCF7 cell lines cell lines upon transfection with miR-493-3p in comparison to miR-controls (Figure S2A). Also the E2F1 mRNA was significantly decreased in HeLa and HCT-116, but not in MCF7 cells (Figure S2A). This may denote a cell line specific difference where in MCF7 cells miR-493-3p induces translational inhibition of E2F1 mRNA without destabilization of the mRNA that instead occurs in HeLa and HCT-116 cells. To better understand the relationship between miR-493-3p induced E2F1 suppression and Mad2 production, we examined the impact of E2F1 RNAi on Mad2. HeLa and HCT-116 cells were transfected with two separate E2F1 targeting siRNAs followed by determination of the E2F1 and Mad2 protein levels 48 hours post-transfection. The results show that despite the significant depletion of E2F1, the Mad2 mRNA and protein levels were as high or higher in the siE2F1 transfected cells than in the control cells transfected with a scrambled siRNA (Figure S2B-D). This suggests that miR-493-3p impairs Mad2 expression directly by targeting Mad2 mRNA while suppression of E2F1 does not contribute to Mad2 depletion.

Earlier, the Mad2 mRNA has been described as an off-target for certain siRNA duplexes targeting ERCC6L (PICH) and Taok1 genes [30, 31]. Therefore we investigated how much the miR-493-3p sequence overlaps with the sequences of these siRNA oligos. Nucleotide blast against the miR-493-3p mature sequence did not reveal any significant sequence similarities with ERCC6L or Taok1 targeting siRNA. Moreover, the miR-493-3p seed sequence is predicted to target Mad2 mRNA at the nucleotides 1230 - 1223, which is not the site of interference of the aforementioned siRNAs.

### Prevention of miR-493-3p targeting of Mad2-3′UTR restores the Mad2 protein levels and resensitizes cells to microtubule drugs

We hypothesized that prevention of interaction between miR-493-3p and Mad2-3′UTR by a synthetic oligonucleotide would reverse the miR-493-3p mediated suppression of Mad2 expression and restore cells’ response to spindle poisons. To test this, we used a target site blocker -oligonucleotide (TSB-MAD2) to compete with the miR-493-3p for the Mad2-3′UTR interference (Figure 2F). First, to validate the efficacy of TSB-MAD2 we co-transfected HeLa cells with different pair-wise combinations of miR-control or miR-493-3p, and a non-targeting TSB-control or TSB-MAD2 followed by determination of the Mad2 protein levels 48 h post-transfection. Quantification of Mad2 Western blots (Figure 2G) indicated that Mad2 protein levels were significantly elevated in cells co-transfected with miR-493-3p and TSB-MAD2 (0.67 +/− 0.19) in comparison to cells transfected with miR-493-3p and TSB-control (0.26 +/− 0.12, *p* = 0.04). We repeated the experiment in the presence of microtubule drugs and time-lapse filmed the cell populations. Analysis of the cell fates showed that the TSB-control did not alleviate the insensitivity of miR-493-3p transfected cells to taxol or nocodazole; 40.6 +/− 10.1% of the taxol treated and 40.6 +/− 8.5% of the nocodazole treated TSB-control and miR-493-3p co-overexpressing cells underwent a forced mitotic exit (Figure 2H, Figure S3). In contrast, cells co-transfected with miR-493-3p and TSB-MAD2 had become more responsive to spindle poisons; only 10.7 +/− 3.2% and 17.0 +/− 1.7% of these mitotic cells exhibited a forced exit from M phase in the presence of taxol and nocodazole, respectively (Figures 2H, Figure S3). We conclude that a specific site within the Mad2-3′UTR facilitates the miR-493-3p targeting, and blockage of this site of interference restores the Mad2 protein levels to a amount that is sufficient to maintain SAC-mediated cell cycle arrest in response to spindle poisons.

### Excess miR-493-3p induces premature sister chromatid separation and aneuploidy in cells

**Figure 3:**
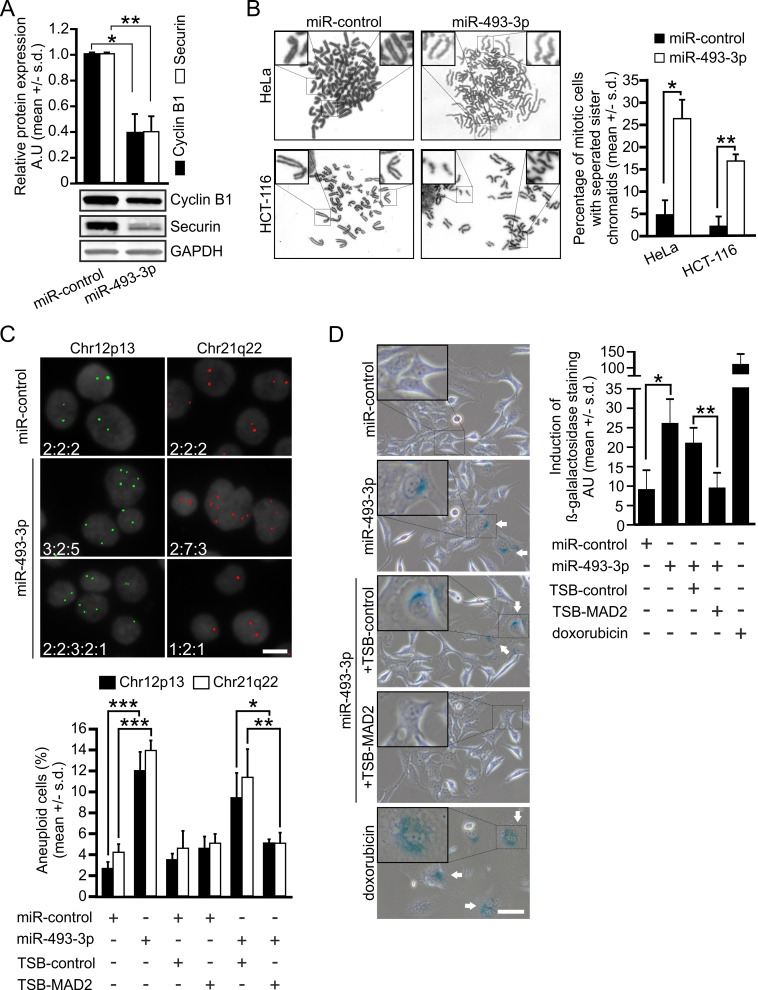
miR-493-3p overexpression leads to aberrant sister chromatid separation and aneuploidy in cells **A.** Quantification of cyclin B1 and securin protein levels from synchronized and M phase arrested miRNA-transfected HeLa cells harvested 12 h after the release from thymidine block. A representative Western blot is shown. **B.** Representative bright field images of metaphase spreads from miR-control and miR-493-3p transfected HeLa and HCT-116 cells. The graph shows quantification of mitotic cells with prematurely separated chromatids (*n* = 150 cells per group). **C.** Representative fluorescence images of DAPI stained HeLa cell nuclei and FISH signals detected for Chr12p13 and Chr21q22. The numbers indicate number of FISH signals scored for each cell in the images. Scale bar, 25 μm. The graph shows frequency of aneuploid cells after the indicated transfections (*n* = 1200 cells per a group and probe). See Table S2 for more information. **D.** Representative micrographs from β-galactosidase assay indicating senescent cells (arrows, *n* = 1500 cells per group, scale bar 50 μm). Doxorubicin served as a positive control. The graph shows the quantification of the signal intensities after the indicated transfections. All data is mean +/− s.d. from 3 independent experiments. The asterisks denote statistical significance (* = *p* ≤ 0.05, ** = *p* ≤ 0.01, *** = *p* ≤ 0.001).

At the molecular level, Mad2 depletion has been linked to premature APC/C activation and precocious degradation of securin and cyclin B1 [32, 33]. These phenomenon replicated in HeLa cells overexpressing miR-493-3p; both proteins were significantly reduced by excess miR-493-3p as compared to controls, measured 12 h after the release of double thymidine block synchronized cells into nocodazole supplemented culture medium (*p* = 0.01, *p* = 0.02, respectively, Figure 3A). Moreover, in chromosome spreads prepared from non-synchronized HeLa and HCT-116 cells a significant change in the frequency of premature sister chromatid separation was observed; 26.3 +/− 4.0% and 16.6 +/− 1.2% of mitotic cells in the miR-493-3p transfected cell populations exhibited separated sister chromatids as compared to 4.6 +/− 3.0% and 2.0 +/− 2.0% in the miR-control transfected cell populations, respectively (HeLa *p* = 0.02, HCT-116 *p* = 0.01, Figure 3B).

Earlier work using Mad2 haplo-insufficient human cancer cell lines and Mad2 overexpressing murine fibroblasts has established a link between Mad2 deregulation and CIN [14, 15]. To test if excess miR-493-3p leads to numerical chromosomal changes, we determined the frequency of aneuploidy in chromosomally stable and near-diploid HCT-116 cell line using Fluorescence *In Situ* Hybridization (FISH). Cells overexpressing miR-control or miR-493-3p were fixed 65 h post-transfection and hybridized with probes for chr12p13 and chr21q22 before enumeration of the chromosome-specific signals in individual interphase cells. Analysis of the pooled results indicated that the miR-493-3p overexpressing cells exhibited a significant increase in the frequency of aneuploidy in comparison to miR-controls; 12.00 +/− 1.80% *vs*. 2.66 +/− 0.57% for probe Chr12p13 and 14.00 +/− 0.86% *vs*. 4.25 +/− 0.75% for probe Chr21q22, respectively (p < 0.001, Figure 3C, Table S2). Moreover, co-transfection of HCT-116 cells with TSB-MAD2 rescued the cells from aneuploidy induced by excess miR-493-3p. In cells co-transfected with miR-493-3p and TSB-MAD2 the total percentage of aneuploid cells was reduced for chr12p13 and chr21q22 probes from 9.41 +/− 2.37% and 11.41 +/− 2.64% to 5.08 +/− 0.28% and 5.08 +/− 0.94% (*p* = 0.02 and *p* = 0.002), respectively, when compared to the miR-493-3p and TSB-control co-transfected cells (Figure 3C, Table S2). We conclude that miR-493-3p-mediated suppression of Mad2 leads to premature separation of sister chromatids and induction of numerical chromosome changes in cultured human cancer cells.

### Overexpression of miR-493-3p causes cellular senescence

Previous studies show that Mad2 depletion contributes to the induction of cellular senescence [34-36]. To examine if overexpression of miR-493-3p has an impact on the frequency of senescence we performed β-galactosidase assay for miRNA transfected HeLa and MCF7 cells. Analysis revealed 3.4-fold average increase of β-gal staining in HeLa cells (*p* = 0.02, Figure 3D) and 4.0-fold in MCF7 cells (Figure S4) by excess miR-493-3p in comparison to the controls. Since miR-493-3p also targets E2F1 and MKK7 [25, 29], both of which are implicated in induction of cellular senescence [37-39], we sought for further evidence of Mad2 contribution on the observed increased senescence in the miR-493-3p overexpressing cells. To this end, we analyzed to what extent the presence of TSB-MAD2 could reduce the elevated cellular senescence induced by excess miR-493-3p. Quantification of the β-gal staining indicated that the average staining intensity was reduced to the same level as in miR-controls; intensity was down by 58 +/− 11% (*p* = 0.004) in cells co-transfected with miR-493-3p and TSB-MAD2 in comparison to cells co-transfected with miR-493-3p and control-TSB (Figure 3D). Based on these results we conclude that suppression of Mad2 protein levels by miR-493-3p increases induction of cellular senescence.

### Introduction of anti-miR-493-3p into cultured cancer cells increases Mad2 protein levels and causes mitotic anomalies

**Figure 4:**
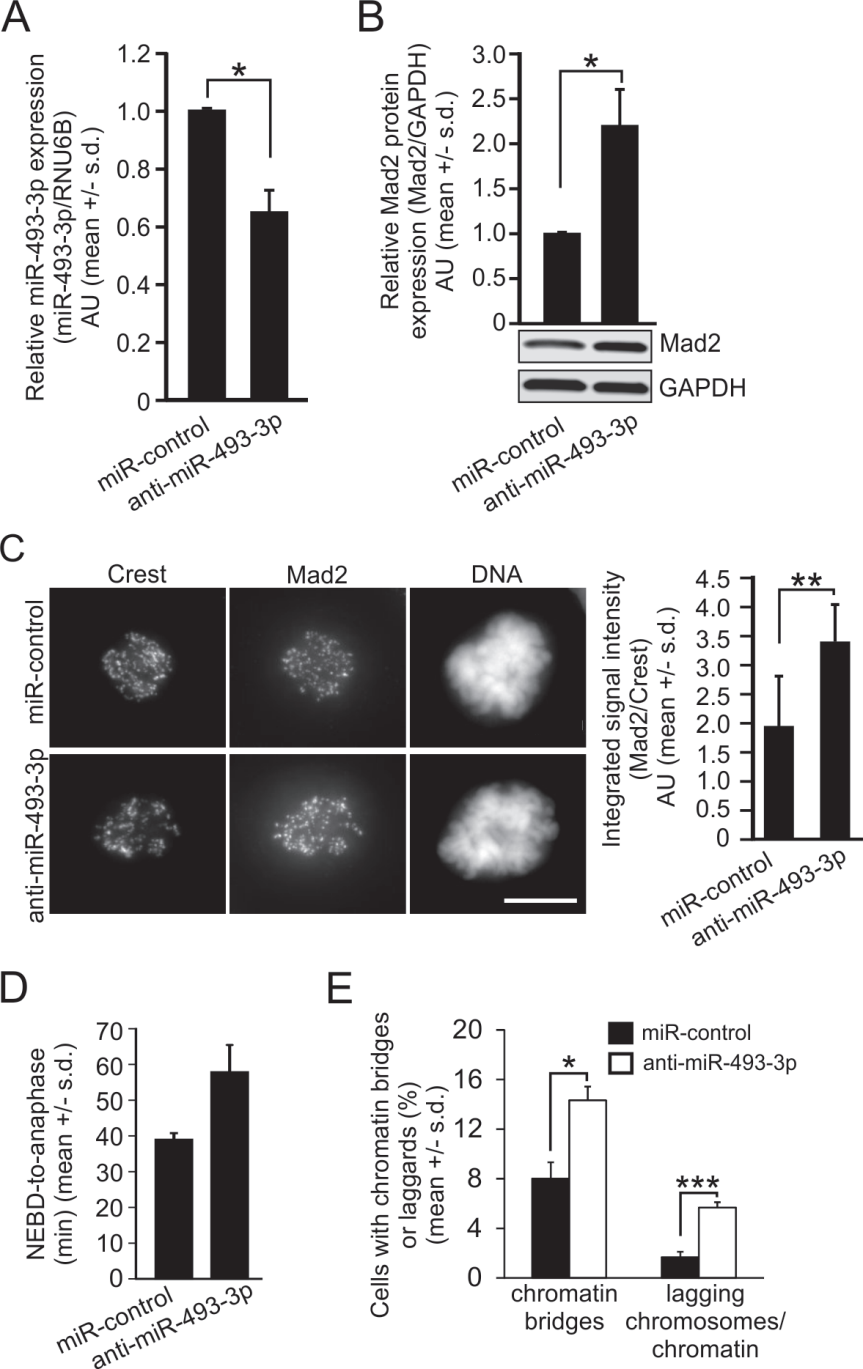
Suppression of endogenous miR-493-3p upregulates Mad2 and induces mitotic anomalies Quantification of the **A.** miR-493-3p and **B.** Mad2 protein levels in HeLa cells 48h after the transfection with miR-control or anti-miR-493-3p. A representative Western blot is shown. **C.** Representative immunofluorescence images showing Mad2 labelling in prometaphase HeLa cells 48 h post-transfection. Crest served as a kinetochore marker and DNA was stained with DAPI. Scale bar, 10 μm. The graph shows quantification of Mad2 signals in whole cells (*n* = 40 mitotic cells per a group). **D.** Quantification of mitotic duration in cells transfected with miR-control or anti-miR-493-3p (*n* = 300 mitotic cells per a group). **E.** The graph shows the quantification of anaphase cells with chromatin bridges and lagging chromosomes/chromatin in miR-control and anti-miR-493 transfected HeLa cells (*n* = 300 anaphase cells per a group). All data is mean +/− s.d. from 3 independent experiments. The asterisks denote statistical significance (* = *p* ≤ 0.05, ** = *p* ≤ 0.01, *** = *p* ≤ 0.001).

Mad2 overexpression has been reported to cause CIN in cells and animals through mitotic errors [15, 40]. Moreover, histological analyses of colon cancer and soft-tissue sarcoma tissue samples have revealed increased frequency of lagging chromatids and anaphase bridges in Mad2 overexpressing cells *in vivo* [41, 42]. To emphasize the effect of miRNA-mediated regulation of Mad2 expression, we transfected HeLa cells with miR-control or anti-miR-493-3p before analysis of the endogenous miR-493-3p and Mad2 levels. qRT-PCR data indicated significant reduction in the endogenous miR-493-3p levels by the anti-miR-493-3p in comparison to miR-controls 48 h post-transfection (Figure 4A). As a consequence, the Mad2 protein levels were an average of 2.2 +/− 0.4 times higher compared to control cells (*p* = 0.03, Figure 4B). Moreover, image-based quantification of Mad2 signal intensities after immunostainings with anti-Mad2 antibodies indicated a significant increase in the amount of Mad2 protein in the individual early mitotic cells overexpressing anti-miR-493-3p in comparison to miR-controls (*p* = 0.009, *n* = 40 cell analyzed per a group, Figure 4C). To evaluate cellular consequences of anti-miR-493-3p induced overexpression of Mad2, we measured the mitotic duration and determined the frequency of anaphase anomalies in anti-miR-493-3p or miR-control overexpressing HeLa cells. The analysis showed that the average duration of mitosis (NEBD-to-anaphase timing) was notably increased by anti-miR-493-3p compared to miR-controls; 57.86 +/− 7.58 min *vs*. 38.98 +/− 1.77 min (Figure 4D). Moreover, microscopic examination of the DAPI-stained cells indicated a significant elevation in the frequency of cells with aberrant anaphase configurations; 14.3 +/− 1.1% of the anti-miR-493-3p transfected cells and 8.0 +/− 1.3% of the miR-control cells exhibited anaphase chromatin bridges (*p* = 0.03), and 5.7 +/− 0.4% of the anti-miR-493-3p transfected cells and 1.7 +/− 0.4% of the miR-control cells exhibited laggards (*p* = 0.001), respectively (Figure 4E). We conclude that miRNA-mediated transient overexpression of Mad2 causes mitotic anomalies.

### Altered expression of endogenous miR-493-3p and Mad2 correlates with aggressive ovarian cancer subtype

**Figure 5:**
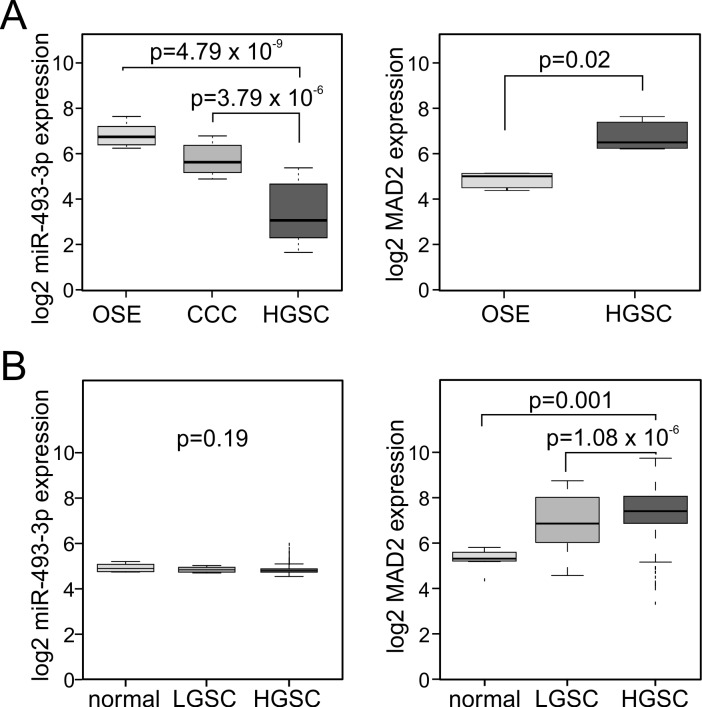
miR-493-3p and Mad2 are inversely expressed in ovarian carcinomas **A.** Box-plots showing the expression levels of miR-493-3p and Mad2 in ovarian surface epithelium (OSE), high-grade serous ovarian carcinomas (HGSC), and for miR-493-3p also in clear cell ovarian carcinomas (CCC), Oslo cohort. **B.** Box-plots showing the expression levels of miR-493-3p and Mad2 in normal ovary tissue (normal), low-grade serous ovarian carcinomas (LGSC) and high-grade serous ovarian carcinomas (HGSC), TCGA cohort.

To understand the functional significance of miR-493-3p dysregulation in cancer we focused on ovarian cancer, a disease often treated with taxane-based chemotherapy. Two separate ovarian cancer sample sets were analyzed retrospectively for miR-493-3p and Mad2 expressions (see materials and methods for the cohort descriptions). In the Oslo cohort an inverse expression of miR-493-3p and Mad2 was observed; miR-493-3p expression was significantly reduced in advanced high-grade serous ovarian carcinoma (HGSC) compared to ovarian surface epithelium (OSE, *p* = 4.79e-09) and clear cell carcinomas (CCC, *p* = 3.79e-06) while Mad2 was significantly elevated in the HGSC *versus* OSE (*p* = 0.02, Figure 5A). Analysis of the ovarian cohort from the Cancer Genome Atlas Consortium (TCGA, [43] confirmed the overexpression of Mad2 in HGSC *versus* low-grade serous ovarian carcinoma (LGSC, *p* = 1.08e−06) and *versus* normal ovarian tissue (*p* = 0.001, Figure 5B). However, in this cohort no significant correlation between miR-493-3p expression and tumor grade was observed (*p* = 0.19, Figure 5B).

**Figure 6:**
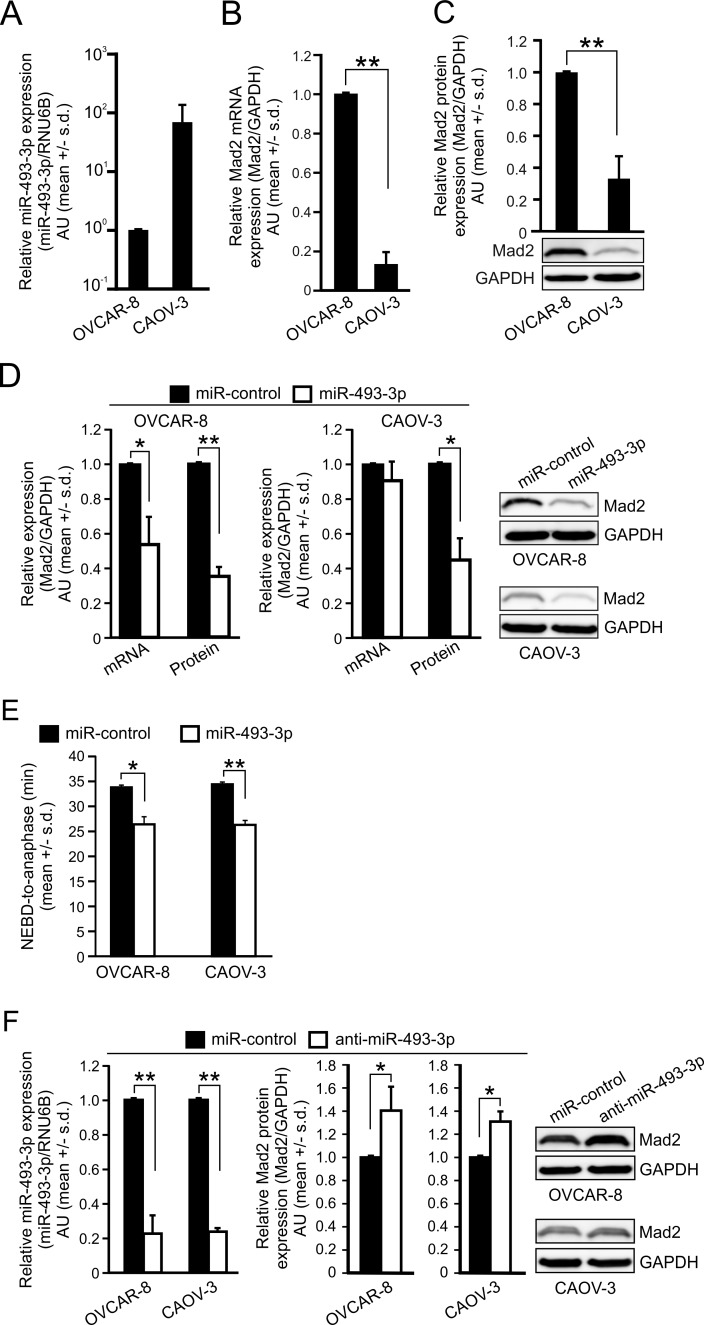
miR-493-3p levels negatively associate with Mad2 gene expression in ovarian cancer cell lines Quantification of **A.** miR-493-3p expression, **B.** Mad2 mRNA and **C.** Mad2 protein in OVCAR-8 and CAOV-3 cell lines. A representative Western blot is shown. **D.** Quantification of Mad2 mRNA and protein levels 48 h after the transfection of OVCAR-8 and CAOV-3 cells with miR-control or miR-493-3p. Representative Western blots are shown. **E.** Quantification of mitotic duration (NEBD-to-anaphase) in OVCAR-8 and CAOV-3 cells transfected with miR-control or miR-493-3p (*n* = 300 cells per group). **F.** Quantification of miR-493-3p and Mad2 protein levels 48 h after the transfection of OVCAR-8 and CAOV-3 cells with miR-control or anti-miR-493-3p. Representative Western blots are shown. All data is mean +/− s.d. from 3 independent experiments. The asterisks denote statistical significance (* = *p* ≤ 0.05, ** = *p* ≤ 0.01, *** = *p* ≤ 0.001).

Next, we examined the relationship between miR-493-3p and Mad2 expressions in two HGSC cell lines, OVCAR-8 and CAOV-3. Analysis of the qRT-PCR and WB readouts indicated that OVCAR-8 cells exhibited low endogenous miR-493-3p expression and high amount of Mad2 mRNA in comparison to the CAOV-3 cells that showed much higher endogenous miR-493-3p expression and reduced Mad2 mRNA (Figure 6A-6B). These significant differences translated to the protein level where the relative amount of Mad2 protein was an average of 66.9 +/− 14.0% lower in the CAOV-3 in comparison to OVCAR-8 (*p* = 0.01, Figure 6C). Next, both cell lines were transfected with miR-control or miR-493-3p followed by measurements of Mad2 mRNA and protein levels. The Mad2 mRNA levels were reduced in the OVCAR-8 cells to 53.9 +/− 15.8% by miR-493-3p overexpression in comparison to controls (*p* = 0.04) while in the CAOV-3 cells having already low amount of Mad2 mRNA no notable further reduction was observed (Figure 6D). However, as in the HeLa, HCT-116, and MCF7 cell lines, excess of miR-493-3p significantly suppressed Mad2 protein levels also in OVCAR-8 and CAOV-3 cells by 65.0 +/− 5.4% (*p* = 0.002) and 55.4 +/− 12.7% (*p* = 0.02), respectively, compared to controls (Figure 6D). It is worth noting that in the CAOV-3 cells only a trace amount of Mad2 protein was observed upon miR-493-3p overexpression. Functionally, overexpression of miR-493-3p significantly accelerated mitosis in both OVCAR-8 (*p* = 0.01) and CAOV-3 (*p* = 0.002) cells (Figure 6E).

Modulation of the endogenous miR-493-3p and Mad2 protein levels in OVCAR-8 and CAOV-3 cells using anti-miR-493-3p resulted in expected changes; the endogenous miR-493-3p was significantly reduced by anti-miR-439-3p in OVCAR-8 (down by 77.4 +/− 10.7%, *p* = 0.006), and CAOV-3 cells (down by 76.3 +/− 2.0%, *p* = 0.01), and Mad2 protein was significantly elevated by 40.5 +/− 20.8% (OVCAR-8, *p* = 0.03) and 31.0 +/− 9.0% (CAOV-3, *p* = 0.03) in comparison to controls (Figure 6F). We conclude that the most aggressive ovarian cancer type *in vivo*, HGSC, is marked by higher expression of Mad2 and lower expression of miR-493-3p in comparison to non-malignant tissue, and that miR-493-3p and Mad2 levels inversely correlate in ovarian cell lines.

### High miR-493-3p expression marks reduced taxol sensitivity in ovarian cancer cell lines and associates with reduced patient survival in ovarian HGSC and in breast cancer post-chemotherapy

**Figure 7:**
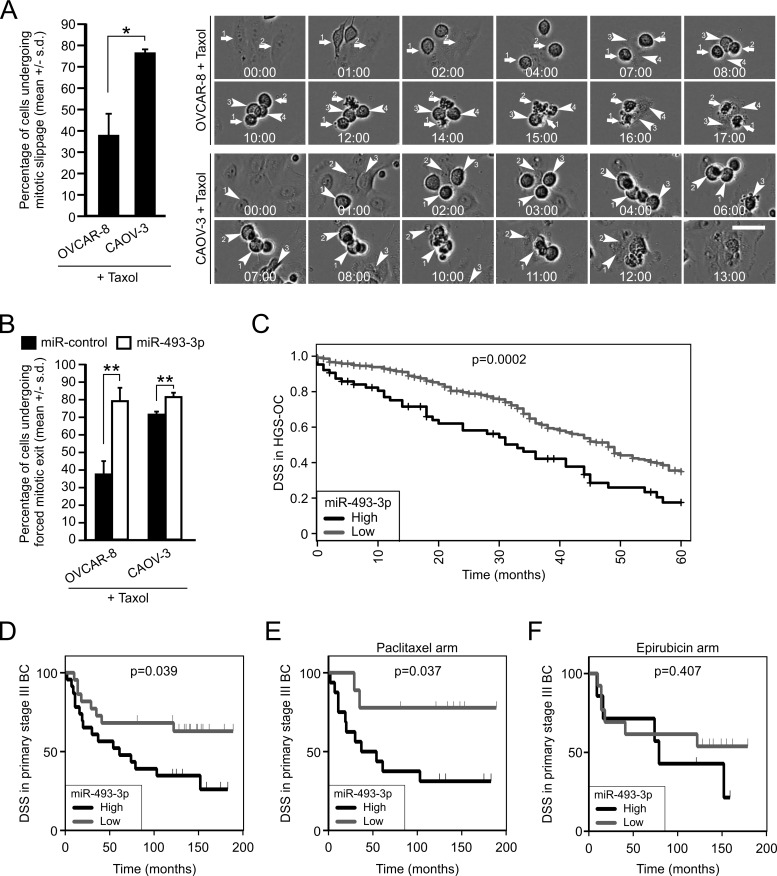
High levels of miR-493-3p confer resistance to taxol *in vitro* and associate with poor survival in ovarian and breast cancer patients with aggressive disease post-chemotherapy **A.** Quantification of mitotic slippage in OVCAR-8 and CAOV-3 cultured in the presence of taxol (*n* = 300 cells). The representative phase contrast still images are from time-lapse films of taxol treated OVCAR-8 and CAOV-3 cell populations showing cells undergoing cell death after prolonged mitotic arrest (arrows) and cells bypassing the taxol block (arrowheads). Scale bar, 50 μm. **B.** Quantification of forced mitotic exit in taxol treated OVCAR-8 and CAOV-3 cells overexpressing miR-control or miR-493-3p. (*n* = 300 cells per group). **C.** The Kaplan-Meier plots representing overall survival of HGSC patients divided into high and low miR-493-3p expression groups. **D.** The Kaplan-Meier plots representing the disease specific survival of primary stage III breast cancer patients divided into high and low miR-493-3p expression and randomized to primary neoadjuvant therapy with either **E.** Paclitaxel or **F.** Epirubicin.

To seek more experimental evidence for the notion that altered miR-493-3p expression contributes to microtubule drug response *via* influencing Mad2 expression, we tested the taxol response of OVCAR-8 and CAOV-3 cells. Both cell lines were observed to possess sub-optimal drug responses; 38.0 +/− 9.5 % of the OVCAR-8 cells and 76.7 +/− 1.2 % of CAOV-3 cells underwent mitotic slippage, respectively (Figure 7A). The difference in the mitotic exit rate between the cell lines was statistically significant (*p* = 0.02), which is in line with the observed divergent Mad2 expression (see Figure 6C). Moreover, when the taxol treated OVCAR-8 and CAOV-3 cell populations were monitored using time-lapse microscopy, the CAOV-3 cells were observed to bypass the mitotic block more rapidly in comparison to OVCAR-8 cells (Figure 7A); the average time spend at taxol block was 8.3+/− 0.8 hour and 4.7 +/− 0.8 hour for OVCAR-8 and CAOV-3 cells, respectively (*p* = 0.02). Next, both cell lines were transfected with miR-control or miR-493-3p to assess the impact on taxol response. Overexpression of miR-493-3p made the cells almost completely insensitive to taxol; during the 24 hour drug incubation 78.7 +/− 7.2% (*p* = 0.005) and 81.0 +/− 2.6% (*p* = 0.003) of miR-493-3p overexpressing OVCAR-8 and CAOV-3 cells, respectively, escaped the taxol imposed M phase block in comparison to miR-controls (Figure 7B). Also the timing of the precocious exit from taxol block was significantly faster compared to controls; the average time spend in taxol arrest by miR-493-3p overexpressing OVCAR-8 and CAOV-3 cells was 3.9 +/− 0.8 hour and 3.0 +/− 0.7 hour while the miR-control cells spend an average of 6.7 +/− 0.8 hour and 4.2 +/− 0.7 hour in taxol block, respectively.

We identified a significant association between high miR-493-3p levels and reduced overall survival (*p* = 0.0023, Figure 7C) in the HGSC patients of the TCGA cohort. Moreover, retrospective analysis of a breast cancer cohort data (Bergen cohort) confirmed the significant association of high miR-493-3p expression with reduced survival in the primary stage III patient group (disease-specific survival, DSS, *p* = 0.0394, Figure 7D). Importantly, data from this breast cancer sub-cohort contained information from two therapeutic arms, having received paclitaxel and the DNA-damaging agent epirubicin, which enabled us to analyze miR-493-3p impact on survival in these chemotherapy groups. Interestingly, in the patients with primary stage III tumors high miR-493-3p expression was found to significantly correlate with lowered DSS in the paclitaxel (*p* = 0.0377) but not in the epirubicin arm (0.4078, Figure 7E-7F). Based on the results we conclude that changed expression of miR-493-3p modulates taxol sensitivity in ovarian cancer cell lines *via* Mad2 depletion, and that high level of miR-493-3p links with poor patient survival in the aggressive forms of ovarian and breast cancer.

## DISCUSSION

Our data reveals a novel miRNA-mediated control mechanism of mitosis, deregulation of which can lead to genomic instability and development of microtubule drug resistance. We establish, for the first time, miR-493-3p as a bona fide negative regulator of Mad2, a critical SAC gene involved in the maintenance of genomic stability. Earlier work has demonstrated that both loss [14, 44] and gain [15] of Mad2 function increase the frequency of mitotic errors and aneuploidy that in animal models stimulate tumorigenesis [14, 15], and affects the recurrence-free survival *in vivo* [18]. Collectively, these results suggest that cells must keep Mad2 expression at optimum to avoid CIN and malignant cell growth triggered by disturbed SAC signaling. One potential regulatory pathway for fine-tuning Mad2 levels, in addition to the normal transcriptional routes, is the miRNA network.

Here we investigated how ectopic modulation of the intracellular amount of miR-493-3p influenced Mad2 expression in a number of human cancer cell lines of different origin. In all the cell lines, changed miR-493-3p expression resulted in significant alterations in Mad2 mRNA and protein levels supporting our notion of an existence of a conserved miR-493-3p-mediated control mechanism of Mad2. Moreover, we showed that post-transcriptional regulation of Mad2 by miR-493-3p is physiologically relevant to faithful cell cycle progression, normal SAC signaling and maintenance of genomic balance. Excess of miR-493-3p resulted in mitotic anomalies and aneuploidy in significant quantities, enabled cells to bypass taxol block, and caused cellular senescence, whereas knockdown of miR-493-3p induced mitotic delay and defective chromosome segregation. These cellular phenotypes are consistent with earlier reports on the consequences of gain- and loss-of-function of Mad2, and support our view of miR-493-3p as a new physiological factor capable of modulating Mad2 levels in human cells. Our *in vivo* data from ovarian cancer is in line with the results of the cell-based assays; the observed inverse correlation between miR-493-3p and Mad2 in ovarian tumors was reproduced under normal endogenous settings in OVCAR-8 and CAOV-3 cell models. Interestingly, loss of miR-493-3p appeared to occur more frequently in HGSC than benign epithelium or less aggressive ovarian cancer forms, which can provide one explanation for the elevated Mad2 levels found in disseminated cancers. It should, however, be noted that since HGSC is derived from fallopian tube epithelia, not the ovary surface, our benign epithelial tissue is not the best possible control for making the normal/tumor pair comparisons. Besides our current results on miR-493-3p, also miR-433-3p [17] and miR-28-5p [45, 46] have recently been reported to target Mad2 mRNA. This underlines the potency of Mad2 regulation by individual miRNAs, whose altered expression can cause mitotic anomalies *via* impairment of Mad2-dependent SAC functions.

miR-493-3p and Mad2 levels are also important in terms of the tumor cells’ sensitivity to microtubule drugs. *In vitro* we demonstrated that excess miR-493-3p impaired cancer cells’ normal response to taxol and nocodazole treatments and enabled them to evade SAC-dependent mitotic arrest and consequent apoptosis due to the reduced Mad2 protein. This proposes existence of an intimate relationship between the endogenous miR-493-3p:Mad2 ratio and taxol efficacy; a cell with low miR-493-3p:Mad2 ratio can maintain taxol imposed mitotic arrest, while a high ratio leads to forced exit from M phase due to precocious inactivation of SAC upon miRNA-mediated Mad2 depletion. We were able to validate this hypothesis in ovarian cancer cell models, in which increased frequency of mitotic slippage was found to correlate with both high endogenous and ectopically modulated miR-493-3p:Mad2 ratio. Besides causing fatigue of SAC, excess miR-493-3p also induced cellular senescence, which as a self-protection mechanism can contribute to the impairment of drug response. Our finding is in line with earlier work by Prencipe et al. who reported increase of senescence upon Mad2 RNAi that was further enhanced by taxol treatment [34]. It should, however, be noted that Mad2 is not the only validated target gene of miR-493-3p implicated in senescence as also E2F1 and MKK7 are involved in the process [25, 29].

Our *in vitro* data suggesting a role for miR-493-3p as one relevant cellular determinant of taxane sensitivity was supported by clinical data from patients with aggressive forms of ovarian or breast cancer. The observed significant correlation between high miR-493-3p levels and reduced patient survival in both HGSC and advanced breast cancer strengthens our hypothesis that decline of Mad2 protein *via* miRNA-mediated mechanisms contributes to tumor recurrence and poor therapeutic outcome. Especially compelling was the finding that survival of stage III breast cancer patients whose tumors showed upregulation of miR-493-3p was significantly reduced in the paclitaxel therapy arm but not in the epirubicin arm. Our data is supported by a recent study (Furlong et al. 2012) in which loss of Mad2 in HGSC correlated with reduced progression-free survival. Moreover, same authors reported that in cell culture Mad2 was depleted by overexpression of miR-433-3p that increased resistance to taxol [17].

Importantly, both miR-493-3p and miR-433-3p locate to the same imprinted gene cluster on human chromosome 14q32 [47]. Changed epigenetic regulation of the locus has been reported in many cancers including melanoma, ovarian cancer, and GISTs [48-50]. This raises a possibility that in cancer, expression of these two Mad2-targeting miRNAs is co-regulated. Hypermethylation of the 14q32 region can drop simultaneously the expression of the two miRNAs, causing a strong and long lasting upregulation of Mad2 to support cancer initiation and advancement of malignant cell growth. On the other hand, demethylation and/or amplification of the 14q32 region can lead to overexpression of miR-493-3p and miR-433-3p, resulting in loss of Mad2 and insensitivity of tumor cells to taxol treatment. Collectively, we propose that altered expression of Mad2-targeting miRNAs modulate SAC signaling, cellular senescence and microtubule drug response, and thereby contribute to the fates of individual cancer cells in the tumor tissue. Future work is expected to show if analysis of Mad2-targeting miRNAs’ expression and/or epigenetic status of the 14q32 locus has diagnostic value in cancer subtyping or in prediction of tumor cells’ sensitivity to clinically utilized microtubule drugs.

## MATERIALS AND METHODS

### Cell culture

HeLa (ATCC) and HCT-116 (Dr. Lauri Aaltonen, University of Helsinki) cells were cultured as described previously [23]. MCF7 (ICLC HTL95021) and CAOV-3 (ATCC) cells were cultured in DMEM (1000 mg/l and 4500mg/l glucose, respectively) supplemented with penicillin/streptomycin (0.1 mg/ml), glutamine (2 mM), sodium pyruvate (1 mM) and 10 % fetal bovine serum. OVCAR-8 cells (DCTD Tumor/Cell Line Repository, NCI) were cultured in RMPI 1640 supplemented with 10% fetal bovine serum, glutamine (2mM) and penicillin/streptomycin (0.1 mg/ml). The cells were incubated at 37°C supplied with 5 % CO2. All cell lines tested negative for mycoplasma after de-freezing.

### Chemicals

Taxol (Paclitaxel, Molecular Probes, Eugene, USA) was used at at 100 nM or 300nM, nocodazole (Sigma, St. Louis, MO, USA) at 150 nM or 300 nM, thymidine (Sigma) at 2 mM, doxorubicin (Sigma) at 50nM. DMSO was used as a negative control.

### Transient transfections

Cell were transfected with Pre-miR™ miRNA Precursors for hsa-miR-493-3p and negative control #1 (Ambion, Thermo Fisher Scientific, Waltham, MA, USA), Anti-miR™ miRNA Inhibitor for hsa-miR-493-3p (Ambion) and with miRCURY LNA™ microRNA Target Site Blockers (Exiqon, Denmark) using Hiperfect (Qiagen, Valencia, CA, USA) according to the manufacturer's protocol for reverse transfection. The sequence of MAD2-TSB was 5′-ATGAAGGTCAAAAGGAGCTA-3′ and for the control-TSB 5′-AGAGCTCCCTTCAATCCAAA-3′. All pre-miRNAs and target site blockers were used at 50nM final concentration. In luciferase assays, pre-miRNAs and plasmids (50 ng) were forward transfected with Lipofectamine 2000 (Invitrogen, Thermo Fisher Scientific). The E2F1 siRNAs used were siE2F1_1: CUCACUGAAUCUGACCACC and siE2F1_2: CAGAUCUCCCUUAAGAGCA at 20nM final concentration.

### Cell cycle synchronization

HeLa cells transfected with miR-control or miR-493-3p were synchronized with double thymidine block as follows. Cells were incubated with the thymidine for 19 h, then released from the S-phase block by washing four times for 15 min with fresh culture medium and incubated for additional 9 h without thymidine, before substituting the culture medium with second dose of thymidine. After 17-35 h incubation with the second thymidine, cells were released into DMSO, taxol or nocodazole.

### Immunoblotting

The method for cell lysis and Western blotting is described elsewhere [23]. The primary antibodies used were mouse anti-Mad2 (Abcam, Cambridge, UK, ab10691, 1:500 or 1:1000), mouse anti-securin (Abcam, ab3305, 1:250), mouse anti-cyclin B1 (BD Biosciences, San Jose, CA, USA, 554178, 1:500), mouse anti-GAPDH (Advanced ImmunoChemical Inc., Long Beach, USA, or HyTest Ltd, Turku, Finland, mAb 6C5, 1:30 000-50 000), and mouse anti-E2F1 (Santa Cruz, Dallas, TX, USA, sc-251, 1:500). Secondary antibodies were Alexa Fluor^®^ anti-mouse 680 (Invitrogen), IR Dye^®^ conjugated anti-mouse 800 (Rockland Immunochemicals Inc., Gilbertsville, PA, USA) and HRP-linked anti-mouse IgG (Cell Signaling Technology). Secondary antibodies were used as 1:5000 dilutions with 1 h incubation at RT. The signal measurement and the quantitative analysis were done using a two channel Odyssey Infrared Imaging System (LI-COR Biotechnology) or ECL detection system and ImageQuant LAS4000 CCD camera (Fujifilm, GE Healthcare).

### Immunofluorescence labeling

For staining of kinetochore proteins, cells on coverslips were pre-extracted with PHEM-buffer (60 mM Pipes, 25 mM Hepes, 10 mM EGTA, 4 mM MgSO4) supplemented with 0.5 % Triton-X-100 for 15 min and after that fixed with 2 % paraformaldehyde in 0.5 % Triton-X-100/PHEM for 15 min. For the whole cell analysis, cells were fixed directly with 2 % paraformaldehyde in 0.5 % Triton-X-100/PHEM for 15 min and then rinsed with MBST (10 mM MOPS, 150 mM NaCl and 0.05% Tween 20). Next the cells were blocked with 20 % boiled normal goat serum (bngs) in MBST for 1h at RT, followed by staining with primary antibodies for overnight at 4°C or 1h at RT. Primary antibodies used were mouse anti-Mad2 (Santa Cruz, Dallas, TX, USA, sc-65492, 1:75), anti-Bub1 (Abcam, ab9000, 1:150) and human autoimmune serum (Crest, Antibodies Incorporated, Davis, USA, 1:200). The secondary antibody dilutions and sample mounting were performed as described elsewhere [51].

### RNA isolation and qRT-PCR analysis

For RNA isolation and cDNA preparation please refer to previous publication [23]. The primers used were Mad2-Fw 5′-CGCGTGCTTTTGTTTGTGT-3′ and Mad2-Rv 5′-GCTGTTGATGCCGAATGAGA-3; and E2F1-Fw 5′-TCCAAGAACCACATCCAGTG-3′ and E2F1-Rv 5′-CTGGGTCAACCCCTCAAG-3′. For GAPDH, the primers used were GAPDH-Fw 5′-AGCCACATCGCTCAGACAC-3′ and GAPDH-Rv 5′-GCCCAATACGACCAAATCC-3′ or GAPDH-Fw2 5′-ACGACCAAATCCGTTGACTC-3′ and GAPDH-Rv2 5′- CTCTGCTCCTCCTGTTCGAC-3′. For measuring miR-493-3p expression, total RNA was isolated with miRVana™ Total RNA Isolation kit (Ambion) and the expression of miR-493-3p was measured with miRNA specific Taqman MicroRNA Assay (#4427975, ID 002364, Applied Biosystems) and Taqman MicroRNA Reverse Transcription Kit (Applied Biosystems). RNU6B was used as an internal control. Taqman analyses were run at the Finnish Microarray and Sequencing Centre (Turku Centre for Biotechnology) with the 7900HT Fast Real-Time PCR System (Applied Biosystems) or with CFX96 Touch™ Real-Time PCR Detection System (Bio-Rad). Results were obtained using the comparative Ct method with SDS 2.4 and RQ manager 1.2.1 softwares (Applied Biosystems) or CFX ManagerTM software (Bio-Rad).

### Luciferase assays

The Mad2-3′UTR firefly luciferase plasmid was used as a reporter plasmid. For the detailed procedure please refer to [51].

### Fluorescence *in situ* hybridization and chromosome spreads

MiRNA overexpressing HCT-116 cells were harvested at 65 h post-transfection and incubated in 0.075 M KCl hypotonic solution at +37°C for 15 min. The cells were fixed using ice cold methanol: acetic acid (3:1). For FISH, the fixative was added to the pelleted cells drop by drop and incubated for 1 h at 4°C, whereas for preparing chromosome spreads the cells were incubated with the fixative for 30min + 20min at RT with gentle swirling to avoid settling of the cells. Later, the cells were pelleted by centrifugation at 200g for 5min and resuspended in fresh fixative. For the slide preparation we used clean glass slide (Superfrost from ThermoScientific) on which the cells were dropped and incubated at RT for 10 min. For FISH with the Vysis LSI ETV6(TEL)/RUNX1(AML1) ES Dual color probe (Abbott Inc, USA) manufacturer's instructions for hybridization and washing steps were followed. ScanR Imaging system (Olympus Corporation, Tokyo, Japan) was used for the image acquisition and analysis. A cell with two FISH signals was considered normal diploid whereas cells with no signal (null), one signal (monosomy), three signals (trisomy), four signals (tetrasomy) or more than four signals (cells that have progressed through two abnormal cell divisions) we scored as aneuploidy. Exceptions to the rule were the cells that showed four signals with both probes; these were considered normal diploid cells at G2 phase. The chromosome spreads were stained using Giemsa's Azure Eosin Methylene Blue solution (Merck KGaA, Darmstadt, Germany).

### Senescence assay

To detect induction of senescence in HeLa and MCF7 cells a Senescence β-galactosidase Staining Kit (Cell Signaling Technology) was used. The cells were fixed and stained 96 h post-transfection using the manufacturer's instructions. Doxorubicin was used as a positive control.

### Image acquisition and analysis

Imaging for IF was performed using the equipment described elsewhere [23]. For live cell imaging we used IncuCyte live-cell imager (Essen Instruments Ltd., Hertfordshire, UK) and Operetta high-content imaging system (PerkinElmer Inc.). Color phase-contrast images for the senescence assay were acquired with Zeiss inverted 200M microscope (Zeiss GmbH, Jena, Germany) equipped with Zeiss AxioCam MRc color camera and AxioVision software (Zeiss GmbH, Jena, Germany). For quantification of the senescence staining, we adjusted the image shadows, midtones and highlights with Adobe Photoshop CS3 (version 10.0.1, Adobe Systems, San Jose, CA, USA) so that only β-galactosidase staining remained visible. The stained areas and staining intensities were measured with Metamorph software version 6.2r6 (Molecular Devices, Downingtown, USA) and normalized to cell number.

### Patient cohorts, tissue specimens and data analyses

Human investigations were performed after approval by an institutional review board and all patient material is managed according to the instructions of ethical committees.

Oslo Cohort: Regarding the material for miR-493-3p and Mad2 expression in ovarian carcinomas and ovarian surface epithelium (OSE), women were enrolled prior to operations for gynecological diseases at Oslo University Hospital during 2003-2012. Tumors comprised primary ovarian carcinoma obtained pre-chemotherapy. OSE samples were collected from patients with benign diseases, as described elsewhere [52]. Tumors were snap-frozen in liquid nitrogen immediately after harvesting, whereas OSE samples were transferred to QiaZol solution (Invitrogen, Carlsbad, CA). All samples were stored at −80°C until processed. The histological classification and clinical staging were according to the World Health Organization classification and FIGO, respectively. Tumors were reviewed by a gynecological pathologist to confirm the histological type and grade, and a frozen section from all biopsies was examined prior to RNA isolation to ensure a tumor component of at least 50% and absence of necrosis. The method for miRNA and mRNA isolation is described elsewhere [53, 54]. Global miRNA expression was analyzed in 12 HGSC, 9 CCC and 9 OSE samples, and global mRNA expression was analyzed in 11 HGSC and 4 OSE samples. The carcinomas for the mRNA analyses were pooled (*n* = 2-3), resulting in 4 HGSC groups [53]. miRNA 2.0 Arrays and Human Genome U133 Plus 2.0 Arrays from Affymetrix (Santa Clara, CA, US) were employed for global miRNA and mRNA expression profiling, respectively. Method and statistical analysis of the global gene expression profiling is described elsewhere [53, 54].

TCGA Cohort: 572 ovarian cancer primary tumor samples and 8 normal ovary tissue samples from the Cancer Genome Atlas Consortium with gene and miRNA expression data available were used in the validation of the association of MAD2 expression with tumor stage and the effects of miR-493-3p expression on patient survival. Gene expression data from Affymetrix U133A arrays were normalized with fRMA [55] and the miRNA expression data (level 3) from Agilent 8x15K miRNA-specific arrays were downloaded from the TCGA repository [43]. Samples with overall survival data available were used for Kaplan-Meier survival analysis with log-rank test implemented in Anduril framework [56].

Bergen Cohort: Regarding the breast carcinoma analysis, out of a cohort of 223 patients diagnosed with locally advanced breast cancer, randomized to primary neoadjuvant monotherapy with either Paclitaxel or Epirubicin, 50 were selected for miRNA profiling based on their response to therapy (25 from each treatment arm), assessed according to the UICC criteria [57]. Patients with a suboptimal response or progressing on first-line treatment were switched to the opposite regimen at the treating physician's discretion. For a detailed description of patients and treatment regime please refer to [58]. Incisional surgical specimens were obtained prior to chemotherapy, and were snap-frozen in the operating theatre. RNA was extracted using mirVana™ kit (Ambion), with RNA quality controlled by Bioanalyzer 2000™.

Library preparation and sequencing were performed at the core facility of the Norwegian Genomics Consortium in Oslo, Norway. 50 bp single-end sequencing was carried out on an Illumina HighSeq 2500 instrument. MiRDeep2 (v.2.0.0.5) was used for post-sequencing analysis, with human reference miRNAs obtained from miRBase (release 21) [59, 60]. Redundant reads mapping to distinct miRNA precursors but identical mature miRNAs were removed. Patients with fewer than 100000 reads mapping to mature miRNA sequences (5 patients) were excluded from analyses. Expression levels of miRNAs were normalized as reads per million mapped (RPMM), and log transformed for statistical analyses.

Kmeans clustering (k = 2) method was used to partition the input data set into clusters in all patient grouping regarding the survival analyses.

### Statistical analysis

Statistical analyses were performed using paired two-tailed Student's t-test, one-way ANOVA or Chi-square test when appropriate. Statistical significance was defined as *P* ≤ 0.05 (*), *P* ≤ 0.01 (**) and *P* ≤ 0.001 (***). Values are presented as the mean ± standard deviation (s.d.).

## SUPPLEMENTAL MATERIAL FIGURES AND TABLES


