# Supplementary material for: Novel Mad2-targeting miR-493-3p controls mitotic fidelity and cancer cells’ sensitivity to paclitaxel
Source: Oncotarget. 2016 Mar 2;7(11):12267–85. doi: 10.18632/oncotarget.7860 (PMC4914283; doi:10.18632/oncotarget.7860)
Supplement: Supplementary file 1 [file oncotarget-07-12267-s001.pdf]

Novel Mad2-targeting miR-493-3p controls mitotic fidelity and cancer cells' sensitivity to paclitaxel

Supplemental Material

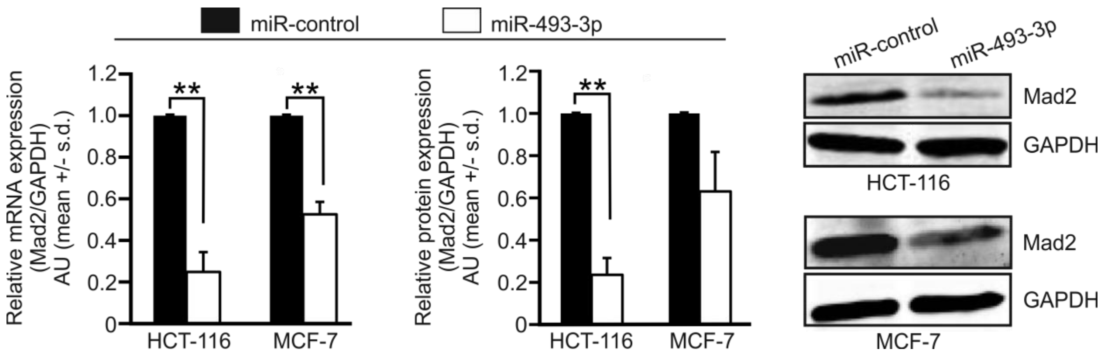

Supplementary Figure 1. Excess miR-493-3p suppresses Mad2 gene expression in HCT-116 and MCF7 cancer cell lines. Quantification of the Mad2 mRNA and protein expression in HCT-116 and MCF7 cells overexpressing miR-control and miR-493-3p. Representative Western blots are shown. All data is mean +/- s.d. from 3 independent experiments. The asterisks denote statistical significance (** = $p \leq 0.01$).

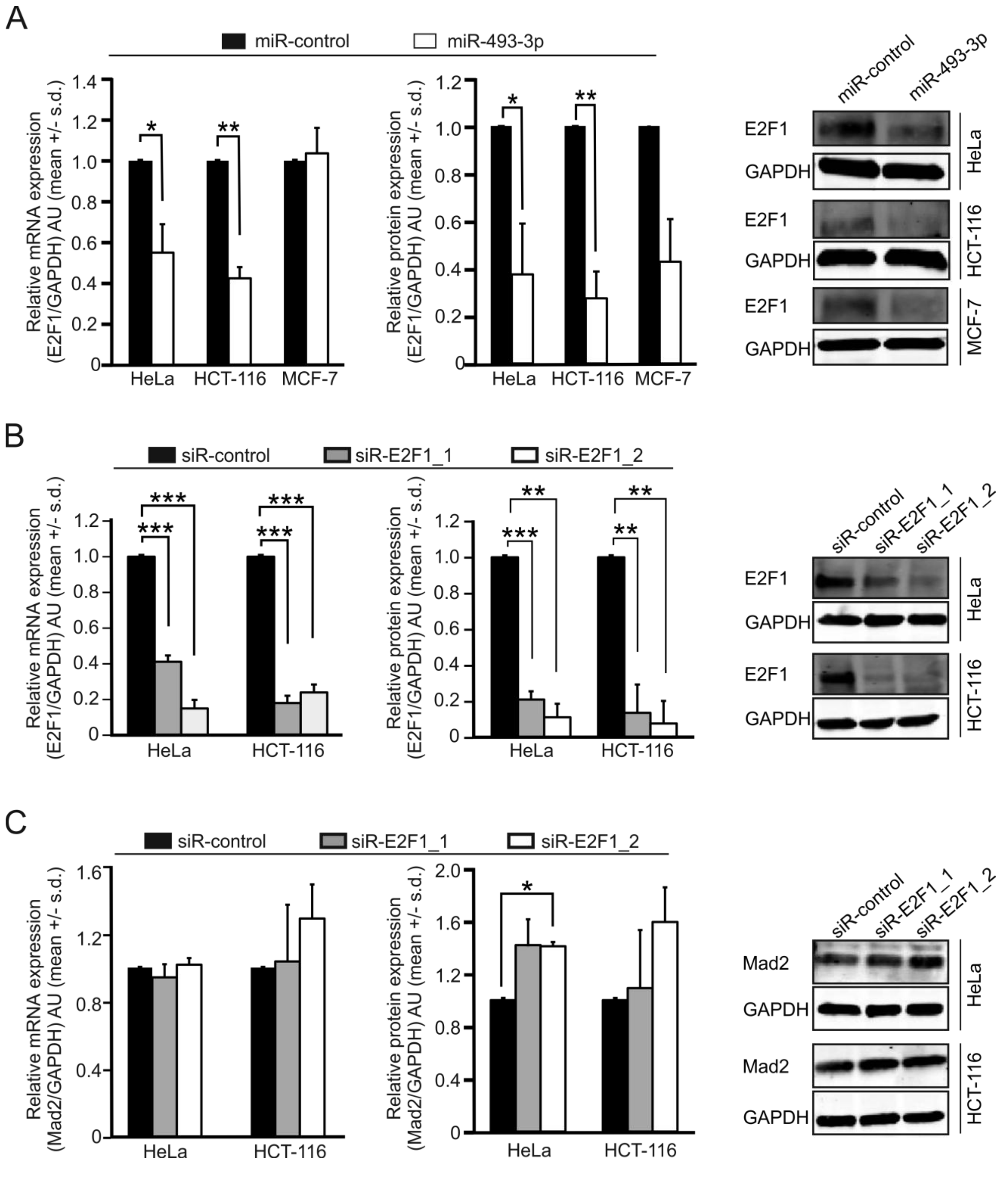

Supplementary Figure 2. miR-493-3p impact on Mad2 expression is not due to E2F1 suppression or off-target effect. miR-493-3p was recently reported to target E2F1 (Gu et al. 2014), which is a putative transcription factor of Mad2 (Hernando et al. 2004). In line with Gu et al (2014), we found that the E2F1 protein levels were significantly reduced in HeLa,

HCT-116 and MCF7 cell lines cell lines upon transfection with miR-493-3p in comparison to miR-controls (**panel A**: quantification of E2F1 mRNA and protein levels in the indicated cell lines overexpressing miR-control or miR-493-3p). Also the E2F1 mRNA was significantly decreased in HeLa and HCT-116, but not in MCF7 cells (panel A). This may denote a cell line specific difference where in MCF7 cells miR-493-3p induces translational inhibition of E2F1 mRNA without destabilization of the mRNA that instead occurs in HeLa and HCT-116 cells. To better understand the relationship between miR-493-3p induced E2F1 suppression and Mad2 production, we examined the impact of E2F1 RNAi on Mad2. HeLa and HCT-116 cells were transfected with two separate E2F1 targeting siRNAs followed by determination of the E2F1 and Mad2 protein levels 48 hours post-transfection. The results show that despite the significant depletion of E2F1 (**panel B**: quantification of E2F1 mRNA and protein in the indicated cell lines transfected with siR-control or E2F1 siRNAs), the Mad2 mRNA and protein levels were as high or higher in the siE2F1 transfected cells than in the control cells transfected with a scrambled siRNA (**panel C**: Quantification of Mad2 mRNA and protein expression in control siRNA or E2F1 siRNA transfected HeLa and HCT116 cells). All data is mean +/- s.d. from 3 independent experiments. The asterisks denote statistical significance (* = $p \leq 0.05$, ** = $p \leq 0.01$, *** = $p \leq 0.001$). Earlier, the Mad2 mRNA has been described as an off-target for certain siRNA duplexes targeting ERCC6L (PICH) and Taok1 genes (Hubner et al. 2010, Westhorpe et al. 2010). Therefore we investigated how much the miR-493-3p sequence overlaps with the sequences of these siRNA oligos. Nucleotide blast against the miR-493-3p mature sequence did not reveal any significant sequence similarities with ERCC6L or Taok1 targeting siRNA (data not shown). Moreover, the miR-493-3p seed sequence is predicted to bind Mad2 mRNA at the nucleotides 1230 - 1223, which is not the site where the aforementioned siRNAs bind in the Mad2 mRNA. Collectively, we conclude

that miR-493-3p impairs Mad2 expression primarily by targeting Mad2 mRNA rather than via suppression of E2F1.

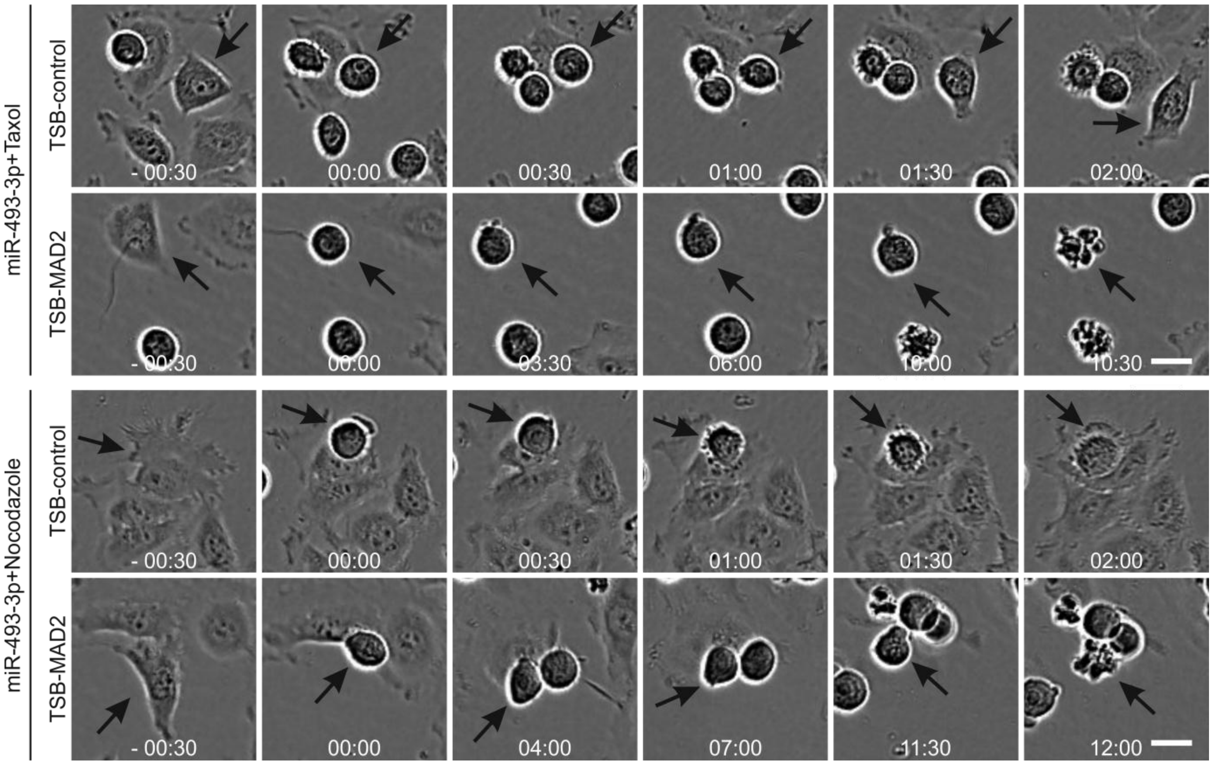

Supplementary Figure 3. Transfection with TSB-MAD2 re-sensitizes miR-493-3p overexpressing HeLa cells to microtubule targeting drugs. Phase contrast images of miR-493-3p overexpressing HeLa cells, co-transfected with TSB-control or TSB-MAD2, cultured in the presence of taxol or nocodazole. The arrows point to individual cells with different fates. Scale bar, 25 µm.

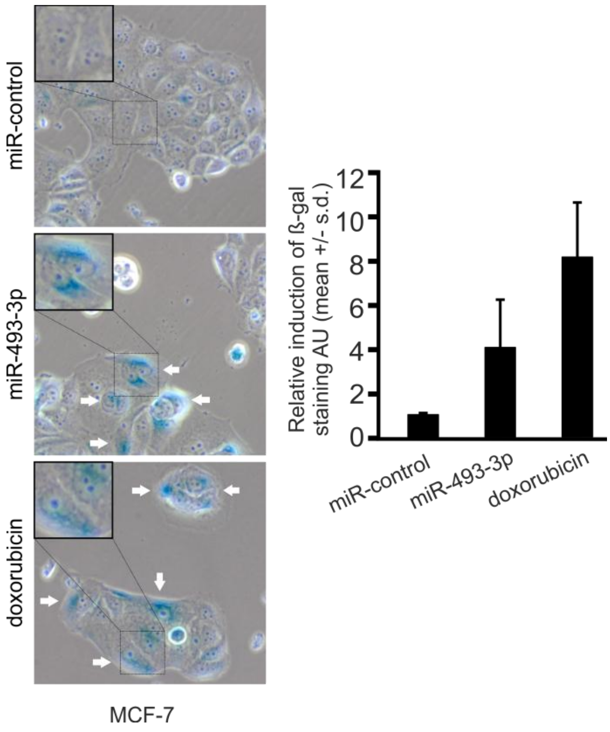

Supplementary Figure 4. Excess miR-493-3p induces senescence in MCF7 cells. Representative images from a senescence assay with MCF7 cells (the arrows point to β-galactosidase positive cells, scale bar 50 µm). The graph shows quantification of β-galactosidase staining (n=1500 cells per group). All data is mean +/- s.d. from 3 independent experiments.

Supplementary Table 1. The 30 most down-regulated genes by excess miR-493-3p in HeLa cells according to illumina microarray analysis.

Gene Symbol	Fold Change	P-value
LOC100132535	-3.13356	2.60E-10
C9orf21	-3.10309	7.45E-11
CERCAM	-2.73788	1.09E-13
DPY30	-2.61869	4.77E-11
DYNLRB1	-2.58263	2.51E-11
RAB7A	-2.46009	7.60E-06
SF3A3	-2.43166	1.12E-09
TOMM5	-2.42967	5.14E-09
B4GALT5	-2.40933	1.24E-10
MRPS24	-2.25362	1.61E-09
NGRN	-2.12242	5.51E-06
LITAF	-2.1083	4.53E-06
GIPC1	-2.06561	6.99E-08
ARL1	-1.96195	1.13E-06
EIF2S3	-1.92113	3.53E-06
KIF3B	-1.86391	4.38E-07
C17orf49	-1.8544	1.11E-06
TXNDC12	-1.78806	6.10E-06
**MAD2L1**	**-1.78328**	**0.000231**
TXLNA	-1.78297	3.17E-06
TPI1	-1.77158	0.000444
CDC45L	-1.75877	4.80E-07
PTGFRN	-1.75736	2.39E-06
H2AFY	-1.74738	0.00018
LOC286016	-1.74672	3.75E-05
CANX	-1.73458	0.000188
ARHGAP23	-1.73253	1.19E-06
PHB	-1.7284	0.00025
ADCY3	-1.71999	2.56E-09
LOC729708	-1.70429	4.89E-06

Supplementary Table 2. Frequency of aneuploidy in miR-control and miR-493-3p overexpressing HCT-116 cells detected using FISH probes for Chr12p13 and Chr21q22.

Chr12p13

Number of FISH Signals \ samples		miR-control	miR-493-3p	miR-control +TSB-control	miR-control +TSB-MAD2	miR-493-3p +TSB-control	miR-493-3p +TSB-MAD2
Two (Diploid)		97.33 +/- 1.80	88.00 +/- 1.80	96.50 +/- 0.50	95.41 +/- 1.04	90.58 +/- 2.37	94.91 +/- 0.28
aneuploid cells	Zero	0.00 +/- 0.00	0.75 +/- 0.25	0.00 +/- 0.00	0.00 +/- 0.00	0.00 +/- 0.00	0.33 +/- 0.28
	One	0.75 +/- 0.25	3.16 +/- 1.62	0.91 +/- 0.52	1.66 +/- 0.62	2.42 +/- 0.76	1.50 +/- 0.43
	Three	1.25 +/- 0.66	5.75 +/- 1.25	2.16 +/- 0.87	2.25 +/- 0.66	5.08 +/- 1.60	2.17 +/- 0.38
	Four	0.58 +/- 0.38	1.25 +/- 0.43	0.17 +/- 0.29	0.58 +/- 0.14	1.00 +/- 0.50	0.83 +/- 0.38
	≥Four	0.08 +/- 0.14	1.08 +/- 0.63	0.25 +/- 0.25	0.08 +/- 0.14	0.92+/- 0.14	0.25 +/- 0.25
Total aneuploid cells		2.66 +/- 0.57	12.00 +/- 1.80	3.50 +/- 0.50	4.58 +/- 1.04	9.41+/- 2.37	5.08 +/- 0.28

Chr21q22

Number of FISH Signals \ samples		miR-control	miR-493-3p	miR-control +TSB-control	miR-control +TSB-MAD2	miR-493-3p +TSB-control	miR-493-3p +TSB-MAD2
Two (Diploid)		95.75+/- 0.75	86.00 +/- 0.86	95.41 +/- 1.66	94.91 +/- 0.80	88.58+/- 2.64	94.91 +/- 0.64
aneuploid cells	Zero	0.00 +/- 0.00	0.83 +/- 0.29	0.00 +/- 0.00	0.25 +/- 0.43	0.17 +/- 0.14	0.17 +/- 0.14
	One	1.58 +/- 0.63	5.17 +/- 1.76	1.92 +/- 0.29	2.00 +/- 0.25	4.67 +/- 0.72	3.00 +/- 0.66
	Three	2.33 +/- 0.29	5.92 +/- 0.63	2.42 +/- 1.46	2.50 +/- 0.50	5.08 +/- 2.02	1.17 +/- 0.14
	Four	0.17 +/- 0.14	1.00 +/- 0.50	0.08 +/- 0.14	0.25 +/- 0.25	0.67 +/- 0.38	0.33 +/- 0.29
	≥Four	0.17 +/- 0.14	1.08 +/- 0.72	0.17 +/- 0.14	0.08 +/- 0.14	0.83+/- 0.38	0.42 +/- 0.14
Total aneuploid cells		4.25 +/- 0.75	14.00 +/- 0.86	4.58 +/- 1.66	5.08 +/- 0.80	11.41+/- 2.64	5.08 +/- 0.94

References for the Supplemental material

1. Gu Y, Cheng Y, Song Y, Zhang Z, Deng M, Wang C, Zheng G, He Z. MicroRNA-493 suppresses tumor growth, invasion and metastasis of lung cancer by regulating E2F1. PloS one. 2014; 9: e102602.

2. Hernando E, Nahle Z, Juan G, Diaz-Rodriguez E, Alaminos M, Hemann M, Michel L, Mittal V, Gerald W, Benezra R, Lowe SW, Cordon-Cardo C. Rb inactivation promotes genomic instability by uncoupling cell cycle progression from mitotic control. Nature. 2004; 430: 797-802.

3. Hubner NC, Wang LH, Kaulich M, Descombes P, Poser I, Nigg EA. Re-examination of siRNA specificity questions role of PICH and Tao1 in the spindle checkpoint and identifies Mad2 as a sensitive target for small RNAs. Chromosoma. 2010; 119: 149-165.

4. Westhorpe FG, Diez MA, Gurden MD, Tighe A, Taylor SS. Re-evaluating the role of Tao1 in the spindle checkpoint. Chromosoma. 2010; 119: 371-379.
